# Enhanced anti-inflammatory activity of chlorogenic acid via folic acid-TPGS-modified liposomes encapsulation: characterization and *In vivo* evaluation on colitis mice

**DOI:** 10.3389/fphar.2024.1437773

**Published:** 2024-08-23

**Authors:** Qing-qing Li, Jia-hui Yan, Zhi-e Zhou, Xiang Geng, Jian-hua Xiong

**Affiliations:** ^1^ College of Food Science and Engineering, Jiangxi Agricultural University, Nanchang, China; ^2^ Key Lab for Agricultural Product Processing and Quality Control of Nanchang City, Nanchang, China

**Keywords:** chlorogenic acid, liposomes, folic acid-TPGS, inflammatory bowel disease, inflammation-related biomarkers

## Abstract

**Introduction:**

Chlorogenic acid (CGA) has been identified to possess salient anti-inflammatory, antioxidant, and anticancer attributes. However, its application is limited by its instability and low bioavailability. Liposomes have been considered effective pharmaceutical delivery vehicles due to their ability to continuously release loaded drugs, improve drug stability, and display good biocompatibility. They can be easily modified by other small molecules to acquire additional biological functions. In this study, we developed and characterized folic acid-TPGS-modified chlorogenic acid liposome (FTCLP) and evaluated its anti-inflammatory activity.

**Methods:**

The successful encapsulation of CGA within FTCLP was confirmed through examination using electron microscopy, fourier-transform infrared spectroscopy (FTIR), and differential scanning calorimetry (DSC). The *in vitro* release characteristics of FTCLP were evaluated using the dialysis bag membrane method. Meanwhile, a dextran sulfate sodium (DSS) -induced colitis model was employed to investigate the anti-inflammatory effect of FTCLP and its mechanism.

**Results:**

The FTCLP exhibited an encapsulation efficiency (EE) of 84.85 ± 1.20% and a drug loading (DL) of 11.67 ± 0.04%. The particle size of FTCLP was determined to be 150.63 ± 0.71 nm, with a polydispersity index (PDI) of 0.198 ± 0.02 and a zeta potential of 2.61 ± 0.38 mV. The *in vitro* release profile followed the Higuchi model, indicating sustained-release characteristics. The *in vivo* study demonstrated that FTCLP treatment was effective in improving the symptoms of DSS-induced inflammatory response, as evidenced by mitigation of weight loss, reduction in the disease activity index (DAI) score, restoration of colon length, and attenuation of colon tissue damage. Furthermore, the levels of pro-inflammatory cytokines, including interferon-gamma (INF-γ), interleukin-1 beta (IL-1β), and interleukin-6 (IL-6), were markedly diminished in both the serum and colon tissue. FTCLP was also observed to suppress the expression of *INF-γ*, *IL-1β*, *IL-6*, tumor necrosis factor-alpha (*TNF-α*), and nuclear factor kappa B (*NF-κB*) *p65*, while concomitantly upregulating the expression of Janus kinase (*JAK*) and signal transducer and activator of transcription 3 (*STAT3*). Besides, the administration of FTCLP was found to result in an increase in the abundance of *Lactobacillaceae* and *Peptostreptococcaceae*, while decreasing the abundance of *Bacteroidaceae*, *Rikenellaceae*, and *Helicobacteraceae*.

**Conclusion:**

Following encapsulation of CGA within liposomes, FTCLP revealed favorable stability and sustained release properties, and enhanced the anti-inflammatory effects by modulating multiple inflammation-related biomarkers. FTCLP has the potential to be a safe and effective drug for targeted therapy of colitis.

## 1 Introduction

Inflammatory bowel disease (IBD), which includes Crohn’s Disease (CD) and Ulcerative Colitis (UC), is a chronic inflammatory disorder of the gastrointestinal tract that affects over 6.8 million individuals globally (Shao et al., 2022). According to the data from the Chinese Center for Disease Control and Prevention, the number of IBD patients in China is expected to exceed 1.5 million by 2025 (Liu et al., 2023). Chronic inflammation in patients with IBD increases the risk of colorectal cancer (CRC) (Shah and Itzkowitz, 2022). The treatment of IBD typically involves medical treatment and surgery. While patients may suffer greater pain and more mental stress during surgery, they are more likely to receive medical treatment. Pharmacotherapy treatments are conventionally administered orally, intravenously, or rectally (Matsuoka et al., 2018), with oral administration being the preferred option for its advantages in medication adherence and patient tolerance (Alqahtani et al., 2021). However, medications used in the treatment of IBD have low tissue targeting and can cause severe side effects, including nausea, diarrhea, and male infertility (Fakhoury et al., 2014; Zhang et al., 2018). Therefore, it is crucial to find an appropriate delivery system that can effectively deliver medication to the affected area and minimize adverse effects, potentially improving treatment outcomes for IBD patients.

Liposomes are a desirable drug delivery system because they have the capacity to enhance pharmacological properties by altering drug pharmacokinetics and biodistribution (Brown and Khan, 2012). They can also release drugs in a sustained manner, enhance drug stability, and demonstrate favorable biocompatibility. Moreover, they are flexible and can be modified by some small molecules in order to acquire enhanced biological capabilities (Yao et al., 2016; Jing et al., 2022).

Folic acid is composed of pteridine, L-glutamic acid, and p-aminobenzoic acid (Tam et al., 2012). It has a high binding affinity for the folate receptor (Zhang et al., 2007), which is overexpressed in damaged colonic epithelial cells, activated colonic immune cells, and colorectal cancer cells (Zhang et al., 2018; Huang et al., 2022). When folic acid is conjugated to compounds or delivery vehicles, it can increase the targeting efficiency, resulting in the focusing of the drug at the folate receptor alpha overexpressed site (Wang et al., 2016; Park et al., 2021; Jing et al., 2022). The utilization of folate-modified liposomes has the potential to reduce the required dosage and the incidence of adverse effects, while simultaneously enhancing the safety and efficacy of drug treatment (Shen et al., 2011; Li et al., 2021; Sanmartín et al., 2021; Jing et al., 2022).

Chlorogenic acid, the major bioactive component of *Lonicerae japonica Thunb*., possesses anti-inflammatory, antiviral, antibacterial, and hypoglycemic activities (Naveed et al., 2018). However, there are some challenges associated with CGA, including its chemical instability, low oral bioavailability, and lack of targeting (Huang et al., 2023). To address these limitations, in this study, we developed a folic acid TPGS (FA-TPGS)-targeted chlorogenic acid liposome that was shown to effectively deliver CGA to the inflamed colon, making it a promising treatment option for IBD. Notably, orally administered CGA encapsulated in FTCLP was more effective than free CGA in treating DSS-induced colitis in mice.

## 2 Materials and methods

### 2.1 Materials

Soybean lecithin (purity> 98%), chlorogenic acid (purity: 98%), and cholesterol (purity: 99%) were obtained from Aladdin Chemistry Co., Ltd. (Shanghai, China). FA-TPGS is purchased from Xian ruixi Biological Technology Co., Ltd. (Xi’an, China). Acetic acid (HPLC) and methanol (HPLC) were obtained from Merck (Darmstadt, Germany). Dextran sulfate sodium (DSS, MW:36000–50000) was bought from Meilunbio (Dalian, China). Phosphate-buffered saline (PBS) was from G-Clone (Beijing, China). Calcium acetate, sodium sulfate, KBr and HCl are all purchased from Xilong Scientific Co., Ltd. (Shantou, China). All solutions were prepared using ultrapure water produced by a Milli-Q Direct 8 Water Purification System.

### 2.2 Synthesis of FTCLP

The preparation of FTCLP was based on Li’s method (Li et al., 2015), with modifications. In brief: soybean lecithin (62 mg), cholesterol (6 mg), and FA-TPGS (3.5 mg) were dissolved in anhydrous ethanol. The organic solvents were then evaporated using a rotary evaporator at 50°C under reduced pressure for 25 min, resulting in the formation of a thin lipid film at the bottom of the flask. Subsequently, the flask was kept under vacuum for an additional 1–2 h to completely remove the remaining traces of solvent. Thereafter, the dry lipid film was hydrated at 60°C for 1.5 h by adding 10 mL of 0.12 mol/L calcium acetate. After sonication using a Scientz-IID ultrasonicator (Ningbo Scientz Biotechnology Co., Ningbo, China), the suspension was transferred to a dialysis bag (MW 8,000–14,000 Da) and dialyzed with sodium sulfate solution for 24 h. Following this, the dialyzed solution was mixed with 11 mL of a 1 mg/mL CGA solution. After incubation in a constant temperature incubator shaker (TS-2102C, Shanghai, China) at 60°C and 180 rpm for 1.5 h, CGA was incorporated into liposomes to obtain FA-TPGS-modified chlorogenic acid liposomes (FTCLP). Blank liposomes without CGA (FTLP) were prepared in the same way by replacing the chlorogenic acid solution with ultrapure water. Both FTCLP and FTLP were filtered through a 0.22-µm pore size filter and stored at 4°C for further analysis. Alternatively, some of them were freeze-dried with sucrose as a lyophilized protective agent for FTIR and DSC analysis.

### 2.3 Characterization of FTCLP

The encapsulation efficiency of FTCLP was quantified using the centrifugal ultrafiltration-HPLC method. Initially, 400 μL of FTCLP was added into a centrifuge tube that was matched with a centrifugal-ultrafiltration tube (Millipore, United States, MWCO = 10 kDa). The mixture was then centrifuged at 12,000 rpm for 30 min at 4°C. The ultrafiltrate was subsequently filtered with a 0.22-μm microporous filter membrane and kept as the non-entrapped CGA sample. Next, 2 mL of methanol was added to 400 μL of another FTCLP, and the mixture was vortexed and sonicated for 10 min to rupture the liposomes and release CGA. The suspension was then centrifuged at 10,000 rpm for 10 min at 4°C and filtered. The filtrate contained both the non-entrapped and entrapped CGA, e.g., total CGA. Afterward, HPLC was employed to quantify the concentration of CGA. A C18 column (Agilent, 4.6 × 150 mm, 4 μm) was used to separate CGA with the mobile phase of methanol/0.2% aqueous acetic acid (75:25, v/v) at a flow rate of 1.0 mL/min. The analysis was performed at 30°C at 326 nm. Finally, EE and DL were calculated using these equations as follows:

$$\begin{array}{l} EE\%=\frac{\text{Total}\;\text{amount}\;\text{of}\;\text{CGA}-\;\text{Amount}\;\text{of}\;\text{non}-\text{entrapped}\;\text{CGA}}{\text{Total}\;\text{amount}\;\text{of}\;\text{CGA}}\\ DL\%=\frac{\text{Total}\;\text{amount}\;\text{of}\;\text{CGA}-\;\text{Amount}\;\text{of}\;\text{non}-\text{entrapped}\;\text{CGA}}{\text{Total}\;\text{weight}\;\text{of}\;\text{Lipids}} \end{array}$$

The morphology of FTCLP was determined using a transmission electron microscope (FEI, United States). The average size, PDI, and zeta potential of FTCLP were measured by dynamic light scattering (DLS) (NanoBrook Omni, Brookhaven, United States). DSC analyses were conducted using a NETZSCH DSC 214 Polyma instrument (NETZSCH, Germany). FTIR spectra were recorded using the KBr method on a Nicolet IS5 FTIR spectrometer (Nicolet, United States).

### 2.4 *In vitro* release of CGA from FTCLP

The *in vitro* release rates of CGA and FTCLP were assessed using the dialysis bag membrane method. The dialysis bags were soaked in double-distilled water for 24 h before use. Encapsulated or non-encapsulated samples (2 mL) were placed in the dialysis bags with the two ends secured with clamps, and then immersed in 200 mL of release medium in an Erlenmeyer flask. The flask was placed in a constant temperature shaking incubator (100 rpm, 37°C). The experiment was carried out in three different release mediums: pH 1.2, 6.8, and 7.4. The samples were initially exposed to a pH of 1.2 (0.1 N HCl) for 2 h. Then, the release medium was replaced with a phosphate buffer at pH 6.8 for a further 2 h, and finally, the pH of the release medium was adjusted to 7.4 to continue the *in vitro* release study for up to 24 h. At specified time intervals, 1 mL of release fluid was withdrawn and replaced with the same volume of fresh release medium. The HPLC method was used to analyze the content of CGA and calculate the cumulative release of free CGA and FTCLP during *in vitro* release. All operations were performed in triplicate. In addition, the *in vitro* drug release data were subjected to a fit using the Higuchi model, which was previously employed to elucidate the drug release behavior (Dian et al., 2014).

### 2.5 Animals and experiment design

C57BL/6 male mice (22 ± 1 g, 7 weeks old) of SPF grade were purchased from Spf (Beijing) Biotechnology Co., Ltd (SCXK (Beijing) 2019–0010, Beijing, China). The animals were fed adaptively for 1 week in a specific pathogen-free animal laboratory (relative humidity, 50%–65%; temperature, 23°C ± 2°C; 12 h/12 h day-night cycle) and had access to food and water 24 h a day. After a 7-day acclimatization period, the mice were randomly divided into a normal control group (n = 6, Control) and a colitis model in mice (n = 36) that were induced with 4% w/v DSS in their drinking water for 7 days (Song et al., 2017). The DSS-treated mice were further divided into six subgroups (n = 6): DSS control group (DSS, ultrapure water), FTLP group (FTLP, 5 mg/kg BW), CGA group (CGA, 5 mg/kg BW), and three groups of FTCLP at different doses as 2.5 mg/kg BW (FTCLP.L), 5 mg/kg BW (FTCLP.M) and 10 mg/kg BW (FTCLP.H). The control group only received normal drinking water throughout the experiment, the other groups were administered the corresponding medication (0.1 mL/10 g BW) by gavage every 12 h, a total of 5 times, after induction. Daily records were kept of body weight, stool blood, and stool consistency, and the DAI was scored according to Table 1 (Hu et al., 2022). The mice were fasted for 12–24 h after the completion of all interventions. Blood samples were taken from the retro-orbital plexus under anesthesia. The serum was collected after centrifugation and stored at −80°C for later analysis. The mice were then sacrificed, and the colon was isolated and measured for its total length. A portion of the colon was taken for hematoxylin-eosin (HE) staining, and the other part was stored at −80°C for future use. Colonic contents were collected and stored at −80°C for analysis. The animal-related study was approved by the Animal Care and Use Committee of Jiangxi Agricultural University (Ethical approval number of experimental animals: JXAULL20230606).

**TABLE 1:** Disease activity index.

Score	Body weight loss%	Stool blood	Stool consistency
0	< 1	negative	stiff, dry, not sticky
1	1–5	slightly positive	stiff, wet, sticky
2	6–10	strong positive	soft, very sticky
3	11–15	slight bloody stool	soft, scattered
4	> 15	bloody stools	loose stool, diarrhea

### 2.6 Colon tissue pathological observation

The colonic tissues were fixed in 4% paraformaldehyde for 24 h, after which the fixed tissue was dehydrated and embedded in paraffin. The tissue was then cut into 4-μm thick sections. Following staining with HE, the sections were observed under a microscope.

### 2.7 Determination of IL-1β, IL-6, and IFN-γ levels in serum and colon tissue

The levels of IL-1β, IL-6, and IFN-γ in serum and colon tissue were determined by ELISA kits, in accordance with the manufacturer’s instructions (Bost Bioengineering Co., Ltd., San Diego, CA, United States).

### 2.8 RT-PCR analysis

Total RNA was isolated from colon tissue according to the manufacturer’s instructions for the TransZol Up Plus RNA Kit (TransGen Biotech, Beijing, China). The concentration and purity of the total RNA were measured using a NanoDrop-300 spectrophotometer (Allsheng, Hangzhou, China). The RNA was then reverse-transcribed into cDNA using the TransScript Uni All-in-One First-Strand cDNA Synthesis SuperMix for qPCR Kit, in accordance with the manufacturer’s instructions, and stored at −80°C for later use. The PerfectStart TM Green qPCR SuperMix (TransGen Biotech, Beijing, China) was used for the qPCR reactions. The reactions were carried out with an initial denaturation at 94°C for 30 s, followed by 45 cycles of denaturation at 94°C for 5 s and annealing at 62°C for 30 s. The mRNA expression levels of *IL-6*, *IL-1β*, *IFN-γ*, *TNF-α*, *NF-κB p65*, *JAK*, and *STAT3* were calculated using the 2^−△△Ct^ method, and data normalization was achieved through the use of the endogenous reference *GAPDH*. The primer sequences used were listed in Table 2.

**TABLE 2:** Primer sequences of RT-qPCR.

Primer name	Forward	Reverse
*GAPDH*	5′-AAT​GTG​TCC​GTC​GTG​GAT​CT-3′	5′-AGA​CAA​CCT​GGT​CCT​CAG​TG-3′
*IL-1β*	5′-AGC​TTC​AAA​TCT​CGC​AGC​AG-3′	5′-TCT​CCA​CAG​CCA​CAA​TGA​GT-3′
*IL-6*	5′-GAC​TGA​TGC​TGG​TGA​CAA​CC-3′	5′-AGA​CAG​GTC​TGT​TGG​GAG​TG-3′
*IFN-γ*	5′-CTG​CTG​ATG​GGA​GGA​GAT​GT-3′	5′-CAC​ATT​CGA​GTG​CTG​TCT​GG-3′
*TNF-α*	5′-CTC​ATG​CAC​CAC​CAT​CAA​GG-3′	5′-ACC​TGA​CCA​CTC​TCC​CTT​TG-3′
*STAT3*	5′-GTT​GGA​GCA​GCA​TCT​TCA​GG-3′	5′-GCA​TGT​CTC​CTT​GGC​TCT​TG-3′
*NF-κB P65*	5′-GGA​GGC​CTT​GAA​GGA​GAT​GT-3′	5′-CAC​ACA​TAG​GTG​CTG​TCT​GC-3′
*JAK*	5′-AAA​GGA​GTC​TGT​GGT​CAG​CA-3′	5′-ACC​AGG​GAC​ACA​AAG​GAC​AA-3′

### 2.9 Gut microbiota analysis

The total DNA extraction and sequencing were completed by Guangdong Magigene Biotechnology Co., Ltd (Guangdong, China). The V3-V4 regions of the bacterial 16S rRNA gene sequences were amplified by PCR using primers: 338F (5′-ACT​CCT​ACG​GGA​GGC​AGC​A-3′) and 806R (5′-GGACTACHVGGGTWTCTAAT-3′), and then the high-throughput sequencing was performed on the Illumina NovaSeq 6000 platform. Unique sequences were subjected to OTU clustering at a 97% similarity threshold. Alpha diversity and beta diversity were used to assess species richness and the similarity of species composition structure, respectively. The abundance of gut microbiota was then evaluated at the phylum and family levels. The Linear Discriminant Analysis Effect Size (LEfSe) was utilized to identify the bacterial biomarkers exhibiting statistical differences, while the linear discriminant analysis (LDA) was employed to illustrate the microbiota with scores exceeding 4. A correlation analysis was conducted at the family taxonomic level. The raw microbial data has been uploaded to the NCBI Sequence Read Archive (SRA) with the accession number PRJNA1139085.

### 2.10 Statistical anaylysis

All experiments were repeated at least three times, and the data were expressed as the mean ± standard deviation. The statistical analysis was performed using IBM SPSS Statistics 24. The one-way analysis of variance (ANOVA) and the Student-Newman-Keul’s (S-N-K) test were employed to compare differences between groups. The level of statistical significance was set at *p* < 0.05. The graphs were plotted using OriginPro 9.1 software (OriginLab Corporation, Northampton, United States).

## 3 Results and discussion

### 3.1 Characterization of FTCLP

**FIGURE 1:**
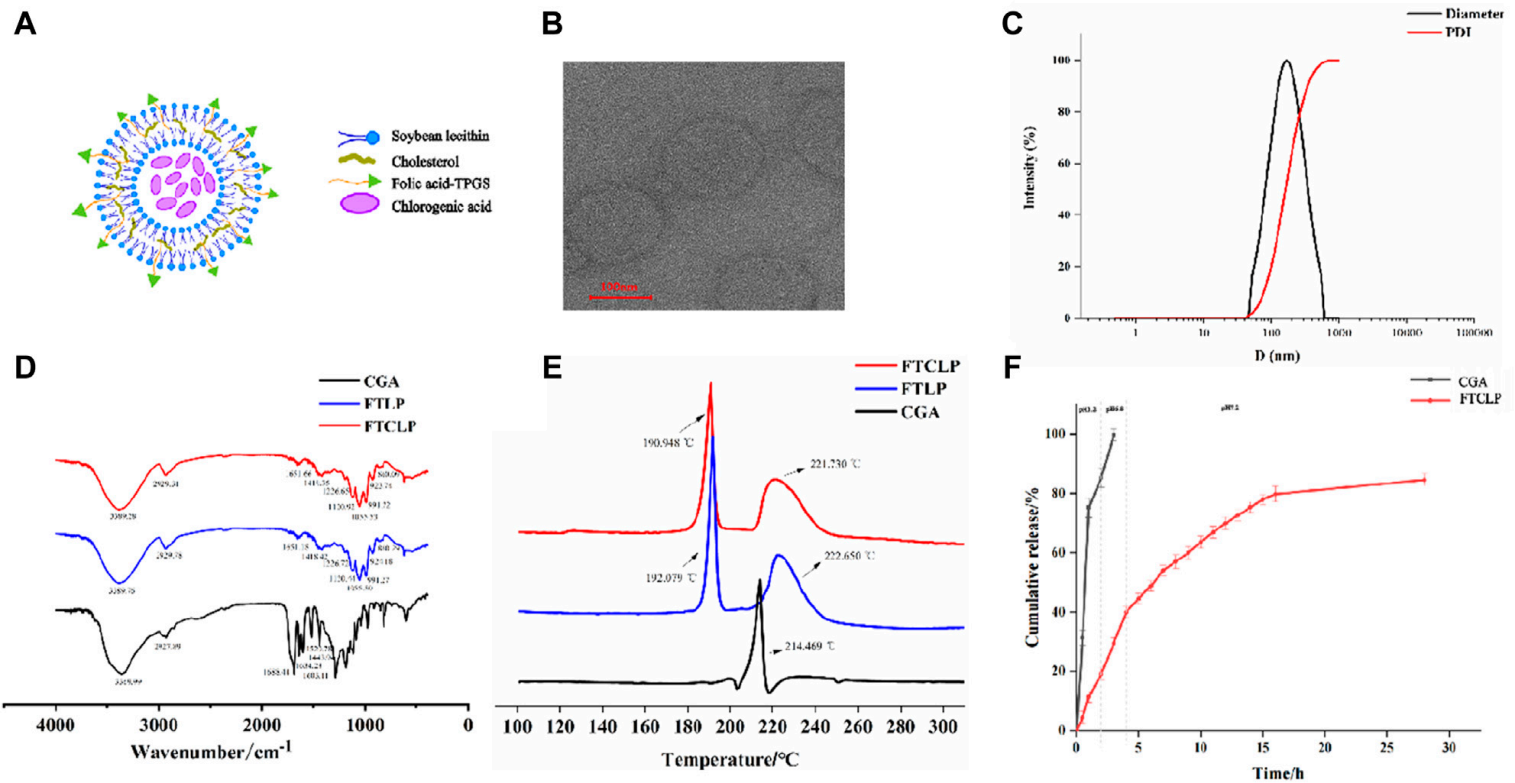
Characterization and *in vitro* release of FTCLP **(A)** The structure of FTCLP **(B)** Transmission electron microscope image of FTCLP **(C)** Size and PDI of FTCLP **(D)** FTIR **(E)** DSC **(F)** *In vitro* release of CGA from FTCLP.

The schematic diagram of FTCLP was shown in Figure 1A, with EE and DL of 84.85% ± 1.20% and 11.67% ± 0.04%, respectively. The transmission electron microscope image of FTCLP in Figure 1B confirmed that the liposome particles were round, uniform, well-dispersed, and small in size (less than 200 nm). The DLS analysis in Figure 1C indicated an average diameter of 150.63 ± 0.71 nm, corroborating the transmission electron microscopy. The PDI was determined to be 0.198 ± 0.02, which fell within an acceptable range of PDI <0.30, implying a homogeneous population of vesicles (Xie et al., 2024). The zeta potential was measured to be 2.61 ± 0.38 mV, which was similar to that of docetaxel-liposomes prepared by Huang et al. (Huang et al., 2007) (2.25 ± 0.2 mV of zeta potential), while docetaxel-liposomes exhibited good stability. The above results suggested that the chlorogenic acid liposomes obtained in this study were small in size, uniformly distributed, and relatively stable.

Samples, including CGA, FTLP, and FTCLP, were subjected to characterization using FTIR spectrophotometry (Figure 1D). The spectrum of liposomes showed peaks at 2,929 cm^−1^ for -CH_2_ asymmetric stretching, 991 cm^−1^ for -CH out-of-plane deformation vibration, and 1,055 cm^−1^ and 1,226 cm^−1^ for symmetric and asymmetric PO^2-^ stretching vibrations, respectively (Meng et al., 2024). O-H/N-H stretching vibrations were indicated by peaks in the range of 3,000–3,700 cm^−1^ with a center frequency of approximately 3,389 cm^−1^, suggesting an overlap between the O-H and N-H vibrational bands (Jakubowska et al., 2020; Wang et al., 2022). Furthermore, the out-of-plane bending vibration of the O-H in the hydroxyl functional group was observed at 923 cm^−1^. Peaks at 1,418 cm^−1^ and 1,651 cm^−1^ signified C-N and carbonyl (-C=O) functional group stretching, respectively (Wang, et al., 2012). These peaks suggested the existence of various functional groups in the liposomes, including phosphodiester, carboxyl, hydroxyl, and amide groups. Amide groups were identified as the characteristic peak of FA-TPGS. Moreover, the wavenumber of 860 cm^−1^ corresponded to the characteristic vibration peak of the double-substituted benzene ring in folic acid (Tang et al., 2023), which was present in both FTLP and FTCLP, demonstrating the retention of the folic acid structure. At the same time, CGA exhibited characteristic band peaks in the 500–2000 cm^−1^ region (Dai et al., 2018) (1,689 cm^−1^ for the stretching vibration absorption of α,β,γ,δ-unsaturated esters; 1,633 cm^−1^ for the stretching vibration absorption peak of alkene C=C; 1,604 cm^−1^, 1,521 cm^−1^, 1,443 cm^−1^ for the vibration of the aromatic ring), but the typical frequencies related to CGA were vanished in FTLP and FTCLP. There was also a slight shift of the characteristic peaks in FTLP and FTCLP. These results revealed that CGA was successfully incorporated into liposomes.

The DSC curves of CGA, TFLP, and FTCLP were shown in Figure 1E. The CGA curve presented a single peak at 214.469°C, corresponding to the melting point of the crystalline region. The FTLP displayed an exothermic peak at 222.65°C attributed to its melting, while the exothermic peak at 192.079°C was caused by the lyophilized protective agent sucrose (Beckett et al., 2006). However, no CGA peak was observed in the DSC curve of FTCLP, while the peaks of FTLP were retained. These results demonstrated that CGA was encapsulated in FTCLP in an amorphous form.

### 3.2 *In vitro* release of CGA from FTCLP

The release profile of liposomes is a crucial factor in predicting their *in vivo* behavior and efficacy (Shao et al., 2017). The drug typically spends 1–2 h in the stomach, 1–6 h in the small intestine, and 1–3 days in the colon (Chu and Traverso, 2022). The *in vitro* release profiles of CGA from FTCLP were investigated in a buffer that underwent a gradual pH change. As shown in Figure 1F, free CGA had a quicker release at pH 1.2 or 6.8 PBS, with 99.70% of CGA being released within the first 3 h. In contrast, FTCLP exhibited a slight initial burst release at pH 1.2 (19.03%), followed by a sustained slower release with a cumulative release of 84.56% at 28 h. The burst release of FTCLP is probably due to disruption of liposomes at low pH. In addition, a linear relationship has been established between the release rate of FTCLP and the square root of time (R^2^ = 0.9522), implying that the release behavior can be explained by the Higuchi model.

### 3.3 FTCLP relieved the intestinal inflammation

#### 3.3.1 FTCLP improved the intestinal inflammation symptoms

**FIGURE 2:**
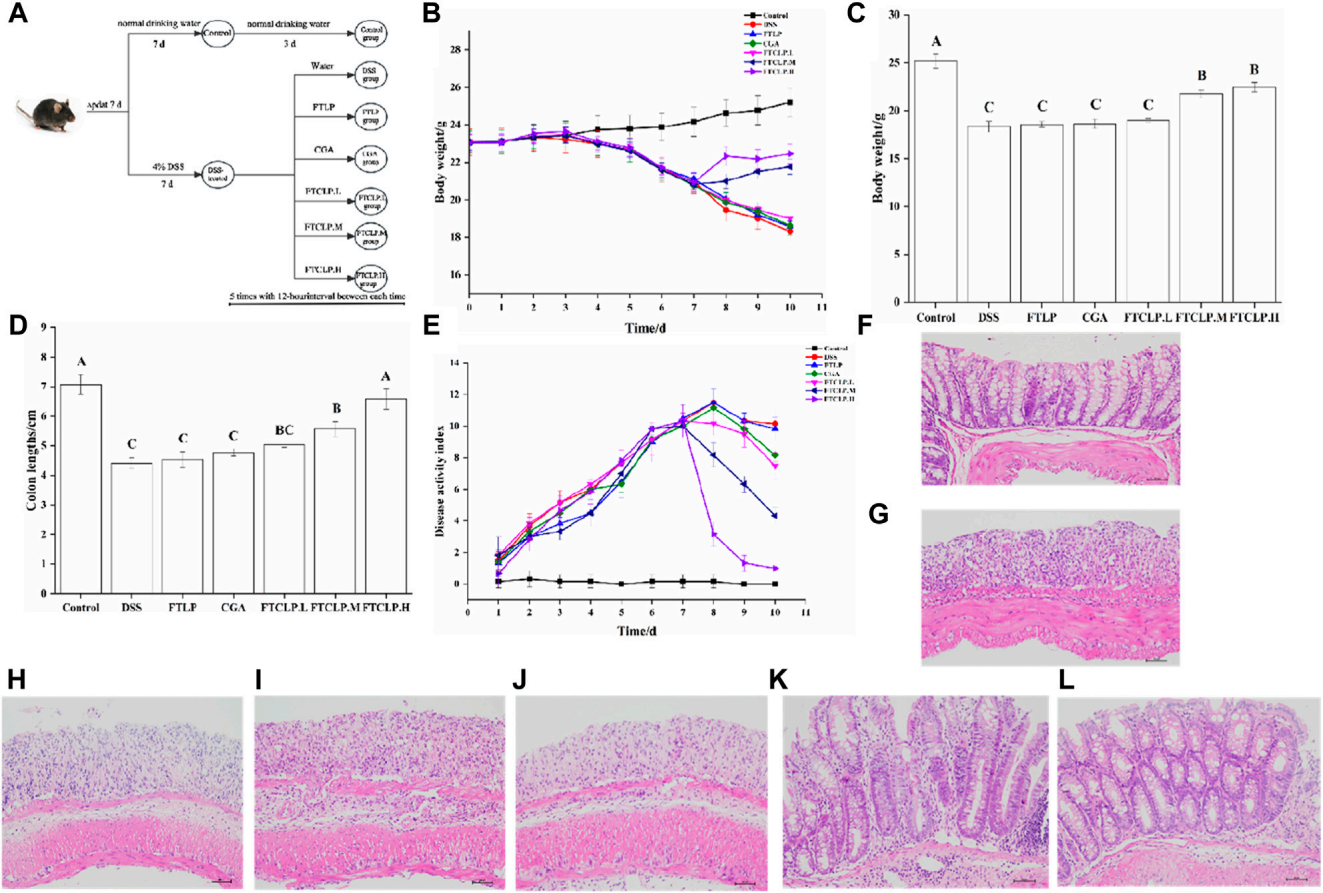
FTCLP improved colitis symptoms **(A)** Schedule of experimental treatments, **(B)** Changes in body weight in mice, **(C)** The body weight of mice at the end of the experiment, **(D)** Colon length, **(E)** DAI score **(F**–**L)** Representative images of mouse colon HE-stained sections: **(F)** Control group, **(G)** DSS group, **(H)** CGA group, **(I)** FTLP group, **(J)** FTCLP.L group, **(K)** FTCLP.M group, **(L)** FTCLP. H group. Values with different uppercase letters are significantly different, *p* < 0.01.

In the study, the induction of a colitis model in mice was followed by the administration of FTLP, CGA, and different concentrations of FTCLP, as shown in Figure 2A. When measuring the change in body weight (Figure 2B), the control group continued to gain weight, while the DSS group showed a sustained decrease in body weight from day 4. The FTLP, CGA, and FTCLP.L groups displayed slightly slower rates of weight loss. Notably, the FTCLP.M and FTCLP.H groups demonstrated a reversion of weight loss tendency and recovery of dry and blood-negative stool samples.

In addition, the body weight of mice at the end of the experiment was depicted in Figure 2C, with the control group having the highest body weight and the DSS group having the lowest. Despite the inability to fully reverse the weight loss, mice in the CGA, FTLP, and FTCLP.L groups demonstrated a reduction in the rapid weight loss induced by DSS. The effect of CGA on DSS-induced colitis was consistent with the results reported by Zhang et al. (Zhang et al., 2019) when using 2% CGA to intervene in DSS-induced colitis in mice. It is worth noting that FTCLP.M and FTCLP.H treatment provided significantly superior effects.

Furthermore, on day 10, the control group displayed the longest colon length, followed by the FTCLP groups (Figure 2D). It was noteworthy that the FTCLP.H group and the control group exhibited no statistically significant difference in colon length (*p* > 0.01). The colon length of mice in the FTCLP.M group was shorter than that of the FTCLP.H group (*p* < 0.01). The colon length of mice in the FTLP group, CGA group, and FTCLP.L group was longer than that of the DSS group (*p* > 0.01). These results indicated that FTCLP was more effective than CGA in reverting the colon length of DSS-induced colitis mice.

Moreover, as illustrated in Figure 2E, the DAI scores of the control group remained stable throughout the whole 10 days. In contrast, the groups exposed to DSS showed a rapid increase in DAI scores, which began to decline after they resumed drinking normal water. The FTCLP intervention facilitated a swift recovery of the DAI scores, in comparison, CGA and FTLP revealed a slower recovery rate in DAI scores.

Besides, in comparison with the control group (Figures 2F–L), the DSS group exhibited significant histological structure damage, marked epithelial breakdown, a disturbed epithelial layer, a pronounced decrease in the number of crypts and goblet cells, and severe infiltration of inflammatory cells. The FTLP, CGA, and FTCLP.L groups failed to restore the damage to the intestinal tissue. Conversely, mice treated with FTCLP.M and FTCLP.H showed a tendency toward normal morphological features in colon tissue, an increase in the number of crypts and goblet cells, and reduced inflammatory cell infiltration. These results identified that FTCLP has the potential to attenuate colon tissue damage.

In summary, neither FTLP nor CGA exhibited complete efficacy in alleviating the symptoms induced by DSS. Nonetheless, FTCLP demonstrated dose-dependent effectiveness in alleviating the symptoms of IBD.

#### 3.3.2 FTCLP decreased the levels of IL-1β, IL-6, and IFN-γ in the serum and colon tissue

**FIGURE 3:**
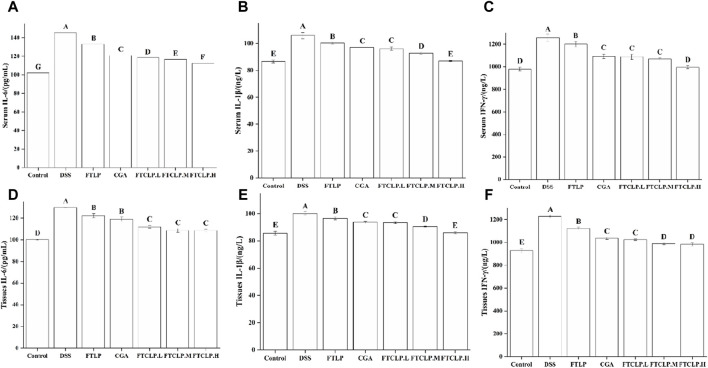
FTCLP decreased the levels of pro-inflammatory cytokines in the serum and colon tissue of mice. **(A**–**C)** Levels of pro-inflammatory cytokines in serum, **(A)** IL-6, **(B)** IL-1β, **(C)** IFN-γ; **(D**–**F)** Levels of pro-inflammatory cytokines in colon tissue, **(D)** IL-6, **(E)** IL-1β, **(F)** IFN-γ. Values with different uppercase letters are significantly different, *p* < 0.01.

Upon ingestion, DSS accumulates in the colon, causing damage to the epithelial barrier and inducing a secondary inflammatory response characterized by the production of pro-inflammatory cytokines, such as IL-1β, IL-6, and IFN-γ (Li et al., 2014). Figure 3 illustrated that the levels of IL-6, IL-1β, and IFN-γ in serum and colon tissue were notably elevated in the DSS group (*p* < 0.01). In contrast, the levels of these cytokines were diminished in the FTLP group, CGA group, and FTCLP group, with the FTLP and CGA groups exhibiting lower levels than the FTCLP group. Specifically, the content of serum IL-1β and IFN-γ showed no significant difference between the control and FTCLP.H groups (*p* > 0.01). Analogously, no significant differences in IL-1β levels were observed between the control and FTCLP.H groups within the colon tissue (*p* > 0.01).

#### 3.3.3 FTCLP modulated gene expression associated with inflammation

To further investigate the underlying molecular mechanisms of how FTCLP alleviates intestinal inflammation, the study analyzed the gene expression levels of markers linked to inflammation, including inflammatory cytokines (*IL-6*, *IL-1β*, *IFN-γ*, and *TNF-α*), kinases (*JAK*), and transcription factors (*NF-κB p65* and *STAT3*).

**FIGURE 4:**
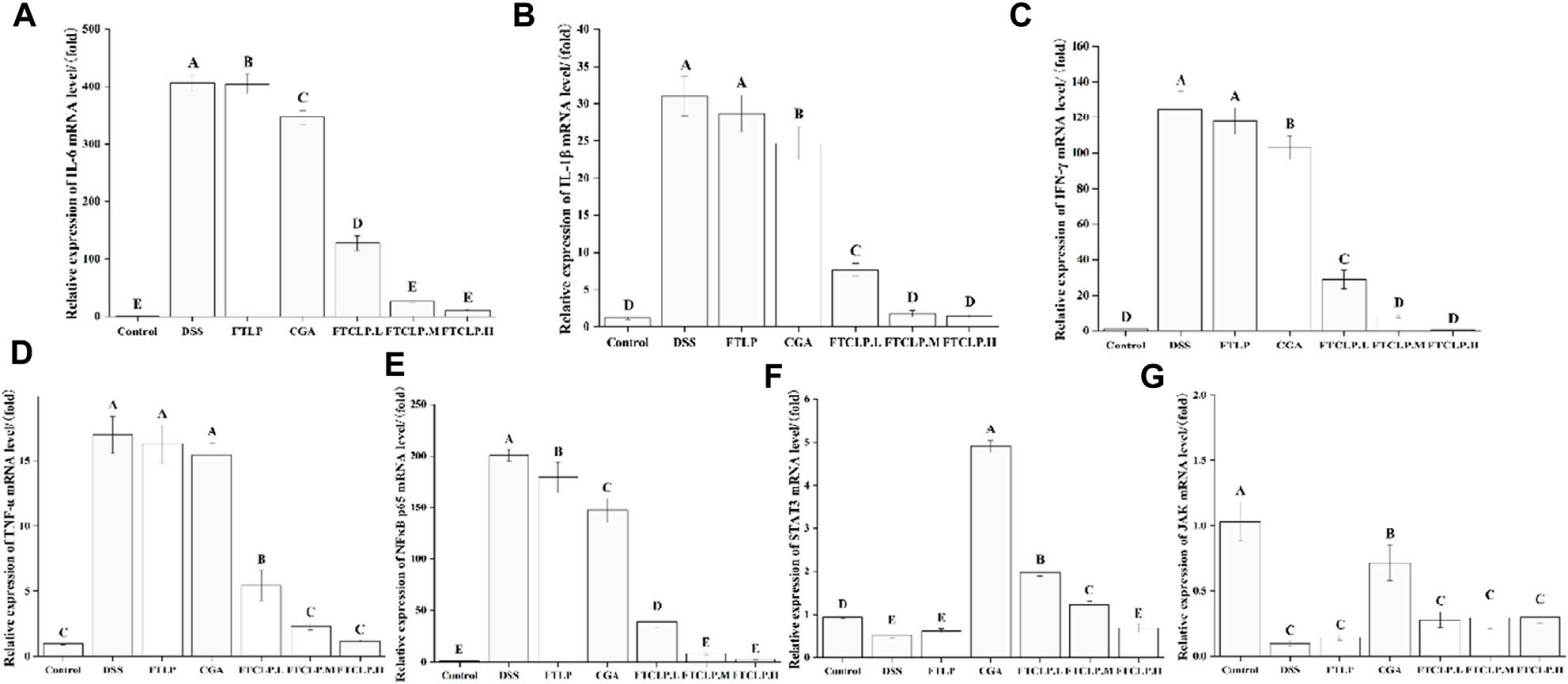
FTCLP modulated gene expression associated with inflammation **(A)** *IL-6*, **(B)** *IL-1*β, **(C)** *IFN-γ*, **(D)** *TNF-*α, **(E)** *NF-κB p65*, **(F)** *STAT3*, **(G)** *JAK*. Values with different uppercase letters are significantly different, *p* < 0.01.

In Figures 4A–D, it can be observed that the mRNA levels of *IL-6*, *IL-1β*, *IFN-γ*, and *TNF-α* were elevated in the DSS group compared to the control group (*p* < 0.01), and their expression was decreased by FTLP, CGA, and FTCLP treatment. In comparison to the DSS group, the FTLP group demonstrated a significant downregulation of *IL-6* mRNA levels (*p* < 0.01), whereas there was no significant reduction in the mRNA expressions of *IL-1β*, *IFN-γ*, and *TNF-α* (*p* > 0.01). In contrast, the CGA group displayed a significant decrease in *IL-6*, *IL-1β*, and *IFN-γ* mRNA levels (*p* < 0.01), with no significant effect on *TNF-α* gene expression (*p* > 0.01). Importantly, the mRNA expression levels of *IL-6*, *IL-1β*, *IFN-γ*, and *TNF-α* exhibited a dose-dependent decrease in the FTCLP group (*p* < 0.01), with no statistically significant difference between the FTCLP.M and FTCLP.H and the control groups (*p* > 0.01).

Furthermore, compared with the control group (Figure 4E), there was a significant upregulation of *NF-κB p65* mRNA expression in the DSS group (*p* < 0.01). However, this upregulation was significantly attenuated in the FTLP group, CGA group, and FTCLP group (*p* < 0.01). Additionally, the mRNA expression of *STAT3* and *JAK* was found to be decreased in the DSS and FTLP groups, while their mRNA expression was increased in the CGA and FTCLP groups to varying degrees (Figures 4F, G). However, the increase in mRNA expression in the FTCLP groups was not as pronounced as that observed in the CGA group. Interestingly, the mRNA expression of *STAT3* in the CGA, FTCLP.L, and FTCLP.M groups was significantly higher than that of the control group (*p* < 0.01).

In the pathophysiology of IBD, aberrant signaling pathways disrupt the regulation of the inflammatory response. Key signaling pathways associated with IBD include the NF-κB, JAK/STAT, PI3K/TLR, and MAPK4 signaling pathways (Guo et al., 2023). NF-κB is a transcription factor that regulates many different genes involved in the development and regulation of inflammatory and immunomodulatory processes (Laurindo et al., 2023). The NF-κB family consists of five members, with the activation primarily initiated by the phosphorylated subunit p65 (Xu et al., 2020). Activation of NF-κB leads to increased transcription of pro-inflammatory mediators like IL-1β, IL-6, TNF-α, and INF-γ, exacerbating intestinal inflammation (Liu et al., 2017; Katsandegwaza et al., 2022). JAK/STAT signaling is highly complex in the inflammatory response. JAKs are responsible for the intracellular signaling of cytokines and are involved in the activation of STAT3 (Cordes F et al., 2020). STAT3 plays a role in IL-10-dependent regulatory functions of Tregs and contributes to the resolution of inflammation (Cordes et al., 2020). However, excessive STAT3 activation promotes intestinal inflammation, including Th17 cell differentiation and suppression of regulatory T cells (Cordes et al., 2020). Studies have shown that CGA inhibits IL-1β-induced proliferation of fibroblast-like synoviocytes by regulating the NF-κB and JAK/STAT pathways, which are commonly used in the treatment of rheumatoid arthritis (Lou et al., 2015). In the study, CGA and FTCLP were found to suppress the gene expression of *INF-γ*, *IL-1β*, *TNF-α*, *IL-6*, and *NF-κB p65*, while increasing the expression of *JAK* and *STAT3*, suggesting that CGA and FTCLP may alleviate inflammatory responses.

#### 3.3.4 FTCLP regulated the abundance and diversity of the gut microbiota

**FIGURE 5:**
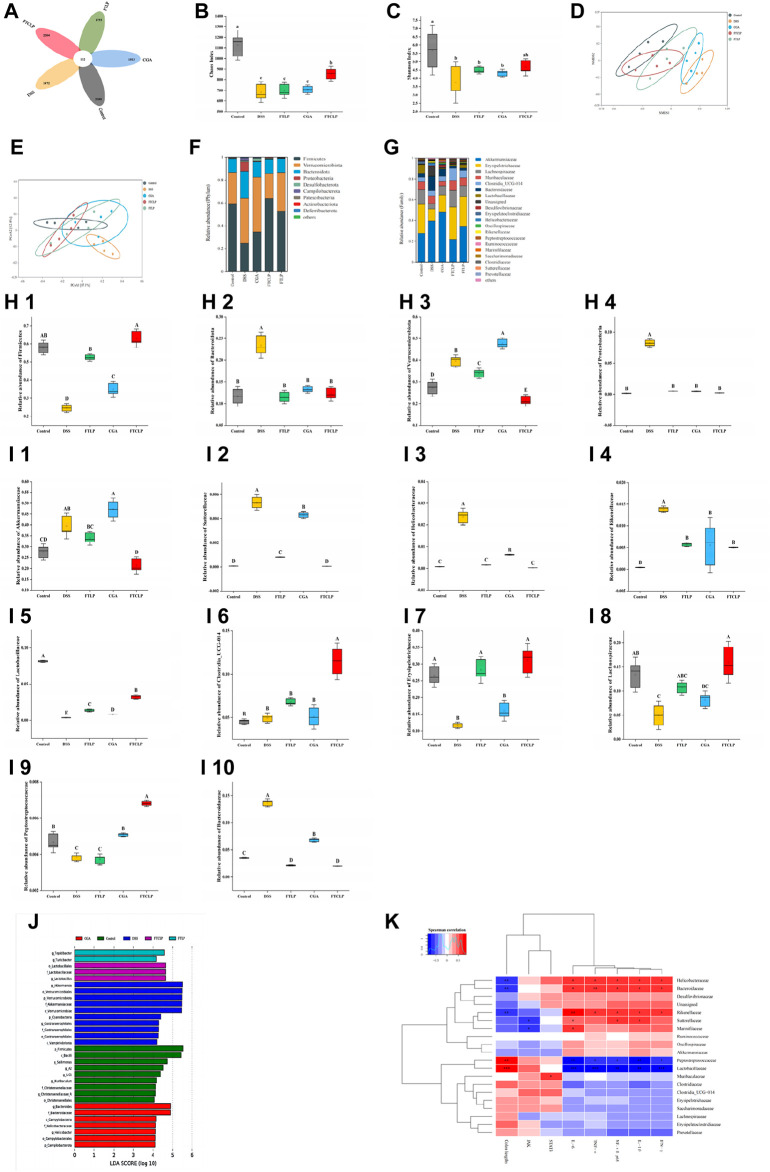
FTCLP regulated the abundance and diversity of gut microbiota. **(A)** Venn diagram, **(B)** Chaos index, **(C)** Shannon index, **(D)** NMDS analysis at the phylum level, **(E)** PCoA analysis at the phylum level, **(F)** phylum-level abundance, **(G)** family-level abundance, **(H)** relative abundance of gut microbiota at the phylum level, **(I)** relative abundance of gut microbiota at the family level, **(J)** LEfSe Analysis (LDA >4), **(K)** Heat map of correlation between related indexes of FTCLP and changes of gut microbiota distribution at the family level. Red color indicates positive correlation, and blue color indicates negative correlation; *p < 0.05, **p < 0.01, and ***p < 0.001. Values with different uppercase letters are significantly different, *p* < 0.01, Lowercase letters indicates *p* < 0.05.

A plethora of evidence highlights that intestinal inflammation is deeply involved in the gut microbiota (Kamada et al., 2013; Zhu et al., 2018; Lobionda et al., 2019). The community composition and abundance of the gut microbiota were characterized by 16S rRNA gene sequencing. As shown in Figure 5A, the number of shared OTUs between the control mice, DSS mice, FTLP mice, CGA mice, and mice treated with a medium-dose FTCLP was 132, while the number of unique OTUs for the five groups was 2,380, 1,472, 1,755, 1,933, and 2,204, respectively. The results showed that the number of species in the DSS group was significantly lower than that in the control group. However, the number of species was partially recovered following the intervention of FTLP, CGA, or FTCLP.

α-Diversity analysis demonstrated that DSS treatment significantly reduced both the Chaos and Shannon indices (Figures 5B, C), whereas FTCLP treatment reversed this effect and resulted in higher indices among intervention groups. There was no significant difference in either of the two indices between the FTLP group, the CGA group, or the DSS group (*p* > 0.05). We then analyzed the similarity of the species composition structure using β-diversity analysis, and as shown in Figures 5D, E, the gut microbiota exhibited significantly different characteristics between the control and DSS groups, reflecting the impairment of DSS on the structure of the gut microbiota. When the DSS group was compared, a significant change in the overall structure of the gut microbiota was observed in the intervention groups. What’s more, the structure of the gut microbiota was most similar between the FTCLP group and the control group. Therefore, the results of the α-diversity and β-diversity analysis indicated that the oral administration of FTCLP ameliorated the disruption and restored the diversity and richness of the gut microbiota.

Species composition analysis and relative abundance (Figures 5F, G) at the phylum level of the gut microbiota revealed that the main bacterial phyla with the highest abundance in the control group were *Firmicutes*, *Verrucomicrobia*, *Bacteroidetes*, and *Proteobacteria*, accounting for about 97.08% of the microbiota. The DSS treatment resulted in a significant increase in the abundance of the phyla *Verrucomicrobia*, *Bacteroidetes*, and *Proteobacteria* (*p* < 0.01) and a significant decrease in the abundance of the phylum *Firmicutes* (*p* < 0.01) compared to the control group (Figure 5H). In comparison to the DSS group, the abundance of the *Firmicutes* phylum was significantly increased in the FTLP group, CGA group, and FTCLP group (*p* < 0.01), while the abundance of the *Bacteroidetes* phylum was significantly decreased (*p* < 0.01). The phylum *Verrucomicrobia* was significantly increased in the CGA group (*p* < 0.01), while it was significantly decreased in the FTLP and FTCLP groups (*p* < 0.01). The abundance of *Proteobacteria* was significantly decreased in the intervention groups (*p* < 0.01), and there was no significant difference between these groups and the control group (*p* > 0.01). IBD patients have been observed to have reduced levels of bacteria with anti-inflammatory capacities and increased levels of bacteria with inflammatory capacities compared to healthy individuals (Nishida et al., 2018). The most consistent change has been a decrease in the relative abundance of *Firmicutes* during inflammation. Studies have reported an increased abundance of the *Proteobacteria* and *Bacteroidetes* phyla in IBD, but a reduction has also been reported (Frank et al., 2007; Walker et al., 2011; Nishida et al., 2018). It is difficult to determine on balance whether the *Bacteroidetes* in the gut have a negative or positive effect on the host. The *Bacteroidetes* contribute to releasing energy from dietary fiber and starch, acting as members of polysaccharide-degrading consortia (Gamage et al., 2017). However, they also participate in the release of toxic products during protein breakdown (Flint and Duncan, 2014; Gamage et al., 2017). It is widely recognized that the *Firmicutes/Bacteroidetes* (F/B) ratio plays a critical role in maintaining normal intestinal homeostasis. A reduced F/B ratio is referred to as dysbiosis and is commonly observed in IBD (Stojanov et al., 2020). The ratios of F/B in the control, DSS, FTLP, CGA, and FTCLP groups were 5.05, 1.04, 4.41, 2.57, and 5.15, respectively. Compared to the control group, the F/B ratio was decreased in the DSS, FTLP, and CGA groups, while the FTCLP group could effectively recover the F/B ratio, suggesting that FTCLP treatment may have a beneficial effect on gut bacteria.

At the family level, the control group is dominated by the *Akkermansiaceae* (27.64%), *Erysipelotrichaceae* (26.58%), and *Lachnospiraceae* (13.38%). Compared with the control group, the abundance of the *Akkermansiaceae*, *Bacteroidaceae*, *Helicobacteraceae*, *Rikenellaceae*, and *Sutterellaceae* was significantly increased in the DSS group (*p* < 0.01), while the abundance of the *Erysipelotrichaceae*, *Lachnospiraceae*, *Lactobacillaceae*, and *Peptostreptococcaceae* was significantly decreased (*p* < 0.01) (Figure 5I). The FTLP, CGA, and FTCLP treatments were found to increase the number of *Erysipelotrichaceae* and *Lactobacillaceae* (*p* < 0.01), while simultaneously decreasing the number of *Bacteroidaceae*, *Helicobacteraceae*, *Rikenellaceae*, and *Sutterellaceae* (*p* < 0.01). In comparison to the FTLP and CGA groups, the relative abundance of the *Erysipelotrichaceae* and *Lactobacillaceae* was higher following the FTCLP intervention, while the relative abundance of the *Helicobacteraceae*, *Rikenellaceae*, and *Sutterellaceae* was lower. In addition, the CGA intervention led to a significant increase in the abundance of the *Peptostreptococcaceae* (*p* < 0.01), while the FTCLP treatment resulted in a significant increase in the abundance of the *Lachnospiraceae*, *Clostridia_UCG-014*, and *Peptostreptococcaceae* (*p* < 0.01), and a significant reduction in the abundance of the *Akkermansiaceae* (*p* < 0.01). These results indicated that FTCLP reversed the dysbiosis of the gut microbiota in mice.

On the one hand, FTCLP reduced the relative abundance of pathogenic bacteria. A very high proportion of the *Akkermansiaceae* may be an indicator of microbial dysbiosis, which is capable of degrading mucin in the gut, causing inflammation and cancer (Grenda et al., 2022). Another indicator of dysbiosis is an increase in the abundance of the *Proteobacteria* phylum, which is associated with various pathogens common to humans and animals (Shin et al., 2015; Liu et al., 2018), the *Sutterellaceae* falls into this category. *Helicobacter pylori*, in the *Helicobacteraceae* family, is also thought to be an important human pathogen, causing a range of gastrointestinal disorders (Malfertheiner et al., 2023). The *Alistipes* (*Rikenellaceae*) is pathogenic in colorectal cancer (Parker et al., 2020).

FTCLP, on the other hand, increased the relative abundance of potentially beneficial bacteria. Probiotic supplementation has been found to improve inflammatory status (Sanchis-Chordà et al., 2019; Ma et al., 2023). Probiotics such as *Lactobacillus* spp. (*Lactobacillaceae*) can modulate JAK/STAT and inflammatory signaling pathways, which have a positive effect on inflammatory responses (Aghamohammad et al., 2022). Clinically, the use of probiotics by UC patients has been demonstrated to prevent flare-ups and inhibit the activation of the transcription factor NF-κB, as well as reducing the expression of TNF-α and IL-1β (Hakansson and Molin, 2011; Huang et al., 2023a). *Clostridia_UCG-014* has been proposed as a potentially beneficial bacteria that positively correlates with the mRNA expression of IL-10 and valeric acid in the colon, which is beneficial for alleviating intestinal inflammation (Wan, 2023). An increase in the proportions of the bacteria *Erysipelotrichaceae* and *Lachnospiraceae*, which are the primary producers of butyrate, was beneficial in attenuating intestinal inflammation. (Li et al., 2020; Wu et al., 2021). *Peptostreptococcus species* (*Peptostreptococcaceae*) have been suggested to exert a positive influence through the synthesis of indoleacrylic acid (a metabolite of tryptophan), enhancing the integrity of the intestinal epithelial barrier and dampening inflammatory reactions (Knuesel and Mohajeri, 2022). As intestinal commensals, *Bacteroides* spp. (*Bacteroidaceae*) play a variety of roles. They can protect other microbial residents from pathogens and provide them with nutrients. However, when the intestinal barrier function is compromised or breached, *B.* spp. can enter normal tissues through the intestinal mucosa and become opportunistic pathogens, which can lead to bacteremia and abscesses in different parts of the body (Thornton et al., 2012; Zafar and Saier, 2021).

LEfSe was employed to further identify specific floras at the phylum to the genus level, and Figure 5J showed the effect size of significantly enriched taxa in each group with LDA >4. The findings indicated that the abundance of *p_Firmicutes* and *c_Bacilli* was higher in the control group. *g_Akkermansia*, *o_Verrucomicrobiaies*, *p_Verrucomicrobiota*, *f_Akkermansiaceae*, and *c_Verrucomicrobiae* expanded substantially and occupied dominantly in the DSS group. The abundance of *g_Tepidibacter* and *g_Turicibacter* in FTLP was significantly elevated. Additionally, the abundance of *g_Bacteroides* and *f_Bacteroidaceae* was increased in the CGA group, while *o_Lactobacillales* and *f_Lactobacillaceae* were enriched in the FTCLP group.

Correlation analysis (Figure 5K) showed that the families *Helicobacteraceae*, *Bacteroidaceae*, *Rikenellaceae*, *Peptostreptococcaceae*, and *Lactobacillaceae* may play a vital role in the exacerbation of colitis. The families *Bacteroidaceae*, *Rikenellaceae*, and *Helicobacteraceae* exhibited a significant positive correlation with the mRNA expression of pro-inflammatory cytokines *TNF-α*, *IFN-γ*, *IL-1β*, *IL-6*, and *NF-κB p65* in the colon, while showing a negative correlation with colon length. In contrast, the *Lactobacillaceae* and *Peptostreptococcaceae* families were positively associated with colon length and inversely correlated with the mRNA expression levels of *TNF-α*, *IFN-γ*, *IL-1β*, *IL-6*, and *NF-κB p65* in the colon. Furthermore, the *Sutterellaceae* family was positively correlated with colonic mRNA expression of *IL-1β*, *IL-6*, and *NF-κB p65*, whereas it was negatively correlated with colonic mRNA expression of *JAK*. A positive correlation was observed between the *Marinifilaceae* family and *IL-6* mRNA expression, along with a negative correlation with the mRNA expression of *JAK* in the colon. The *Muribaculaceae* family showed a positive correlation with the mRNA expression of *STAT3* in the colon. These findings suggested that the composition and abundance of gut microbiota were correlated with gut inflammation. By increasing the abundance of *Lactobacillaceae* and *Peptostreptococcaceae*, and decreasing the abundance of *Bacteroidaceae*, *Rikenellaceae*, and *Helicobacteraceae*, FTCLP effectively modulated the gut microbiota to mitigate the inflammation response.

Studies have shown that upon oral administration, a portion of CGA is immediately absorbed in the stomach and small intestine in its original form, undergoes metabolism in the liver, while the remaining portion enters the cecum and colon (Li et al., 2015b; Zatorski et al., 2015). In the colon, CGA is hydrolyzed and metabolized by esterase and intestinal flora on the intestinal mucosa (Li et al., 2015b). However, after liposomal encapsulation, FTCLP demonstrates robust resistance to gastric acid, allowing a higher percentage of FTCLP to reach the intestines. Folic acid on the surface of liposomes exhibits targeted functionality, accumulating at inflammatory sites (Wang et al., 2016). More importantly, the good biocompatibility of liposomal membranes enables them to penetrate cell membranes and biological barriers (Yao et al., 2017). This allows them to enter inflammatory cells, where they facilitate a controlled release of CGA within the targeted cellular environment, resulting in a prolongation of the CGA residence time at the disease site, thereby enhancing its pharmacological activity and maximizing therapeutic efficacy.

## 4 Conclusion

In summary, this study successfully synthesized and characterized FTCLP, and confirmed that encapsulation within folic acid-TPGS-modified liposomes resulted in an improvement of the anti-inflammatory properties of CGA. *In vitro* studies have demonstrated that FTCLP possesses favorable stability and sustained release properties. The *in vivo* results indicated that, in comparison with the free CGA, FTCLP exhibited an improvement in the symptoms of the DSS-induced inflammatory response. Moreover, the levels of serum and colonic tissue pro-inflammatory cytokines were significantly reduced, and the gene expression associated with inflammation was modulated. Additionally, the dysregulated gut microbiota was improved after the administration of FTCLP. These findings suggest that FTCLP may be a viable option for the practical and effective management of IBD.

## Data Availability

The data presented in the study are deposited in the NCBI Sequence Read Archive (SRA) repository, accession number PRJNA1139085.
